# SWATH-based proteomics identified carbonic anhydrase 2 as a potential diagnosis biomarker for nasopharyngeal carcinoma

**DOI:** 10.1038/srep41191

**Published:** 2017-01-24

**Authors:** Yanzhang Luo, Tin Seak Mok, Xiuxian Lin, Wanling Zhang, Yizhi Cui, Jiahui Guo, Xing Chen, Tao Zhang, Tong Wang

**Affiliations:** 1 Key Laboratory of Functional Protein Research of Guangdong Higher Education Institutes, Institute of Life and Health Engineering, College of Life Science and Technology, Jinan University, Guangzhou 510632, P. R. China; 2 Department of Otorhinolaryngology, The First Affiliated Hospital, Jinan University, Guangzhou 510632, P. R. China.

## Abstract

Nasopharyngeal carcinoma (NPC) is a serious threat to public health, and the biomarker discovery is of urgent needs. The data-independent mode (DIA) based sequential window acquisition of all theoretical fragment-ion spectra (SWATH) mass spectrometry (MS) has been proved to be precise in protein quantitation and efficient for cancer biomarker researches. In this study, we performed the first SWATH-MS analysis comparing the NPC and normal tissues. Spike-in stable isotope labeling by amino acids in cell culture (super-SILAC) MS was used as a shotgun reference. We identified and quantified 1414 proteins across all SWATH-MS analyses. We found that SWATH-MS had a unique feature to preferentially detect proteins with smaller molecular weights than either super-SILAC MS or human proteome background. With SWATH-MS, 29 significant differentially express proteins (DEPs) were identified. Among them, carbonic anhydrase 2 (CA2) was selected for further validation per novelty, MS quality and other supporting rationale. With the tissue microarray analysis, we found that CA2 had an AUC of 0.94 in differentiating NPC from normal tissue samples. In conclusion, SWATH-MS has unique features in proteome analysis, and it leads to the identification of CA2 as a potentially new diagnostic biomarker for NPC.

Although nasopharyngeal carcinoma (NPC) is a rare cancer type in most parts of the world, it is a common malignancy in a few well-defined populations, including natives of Southern China, Southeast Asia, the Middle East and Southern Africa^1^. In Guangdong province, China, the approximate incidence rate is 10~30 cases per 100000 people-years^2^, which positions NPC one of the most serious local health treats. Similar to other cancer types, the five-year overall survival (OS) of NPC decreases along with the increased disease stages. For example, Lee *et al*. have found that the OS according to stage is 90% for stage I, 84% for stage II, 75% for stage III, and 58% for stage IVA-B^3^. To be noted, a very promising recent clinical trial has reached a 3-year failure-free survival of 80% in the late stage NPC treatment^4^. Despite this, it has been generally accepted that early diagnosis has critical significance on containing the NPC disease progression and increasing survival, which emphasizes the field of NPC biomarker discovery.

As NPC is highly correlated to the Epstein-Barr virus (EBV) infection, EBV viral RNAs (miRNAs^5^,^6^, long non-coding RNAs^7^) and proteins are of diagnostic powers. In clinical practice, EBV capsid antigen IgA (VCA-IgA), early antigen Ig A (EA-IgA), nuclear antigen (EBNA1) IgA, and circulating EBV DNA are widely used for diagnosis and screening of NPC^8^,^9^,^10^. It is known that EBV infection is very common in general population; thus, the viral factor in peripheral blood is not a necessary indicator of NPC, rather an efficient parameter to monitor the NPC progression or therapeutic effects^11^.

As such, the host protein factors are emphasized for their biomarker potentials. Individual proteins, such as Bmi-1^12^, HMGB1^13^ and KLHDC4^14^, have been associated with diagnosis and/or prognosis of NPC. Furthermore, high-throughput methods, including proteomics and next generation sequencing, are rapidly moving the field forward^15^. For example, Chen *et al*. employed isobaric tags for relative and absolute quantitation (iTRAQ) -based mass spectrometry (MS) to compare the motile and non-motile NPC cells, in which they found and validated the biomarker potentials of RAN, SQSTM1 and TRIM29^16^. Besides iTRAQ-MS, shotgun MS approaches are primarily based on the data-dependent acquisition (DDA) mode, known with the advantage of deep proteome coverage. Theoretically, shotgun MS is a probability based identification; and controlling the false discovery is an inevitable challenge, especially when dealing with the database search against large reference databases^17^.

As a major advance in the field of proteomics, the data-independent acquisition (DIA) based approach of sequential window acquisition of all theoretical fragment-ion spectra (SWATH) -MS has brought general attentions^18^. The basic theory of SWATH is to acquire all theoretical fragment ion spectra, and assemble them back to the parent ions to achieve high-throughput identification and quantification^19^. This is an extraordinary and complementary expansion of the relatively low-throughput selected/multiple reaction monitoring (SRM/MRM)^20^,^21^.

SWATH has been justified to be valuable in a few cancer types for biomarker discovery, such as prostate cancer^22^, colorectal cancer^23^, gastric cancer^24^, and lung cancer^25^. However, its application in the NPC field is very limited. In this study, we performed the first SWATH-MS analysis comparing the NPC and normal tissues, in which we successfully identified and verified carbonic anhydrase 2 (CA2) as a potentially new diagnosis biomarker of NPC.

## Results

### SWATH and super-SILAC MS analyses

To quantify the proteome change of NPC tissues, we took advantage of the DIA feature of SWATH, and the super-SILAC MS was used as a general shotgun MS verification. Tissue lysates from 9 normal and 9 NPC subjects were respectively pooled and analyzed by super-SILAC MS analysis. In addition, 6 normal and 5 NPC subjects from the same batch of donors used for the super-SILAC MS were individually analyzed with SWATH-MS. Six more clinical samples were obtained for the subsequent verification steps. No statistical difference was observed regarding donor gender or age (*P* > 0.05, Fisher’s exact test, Supplementary Table S1).

**Figure 1:**
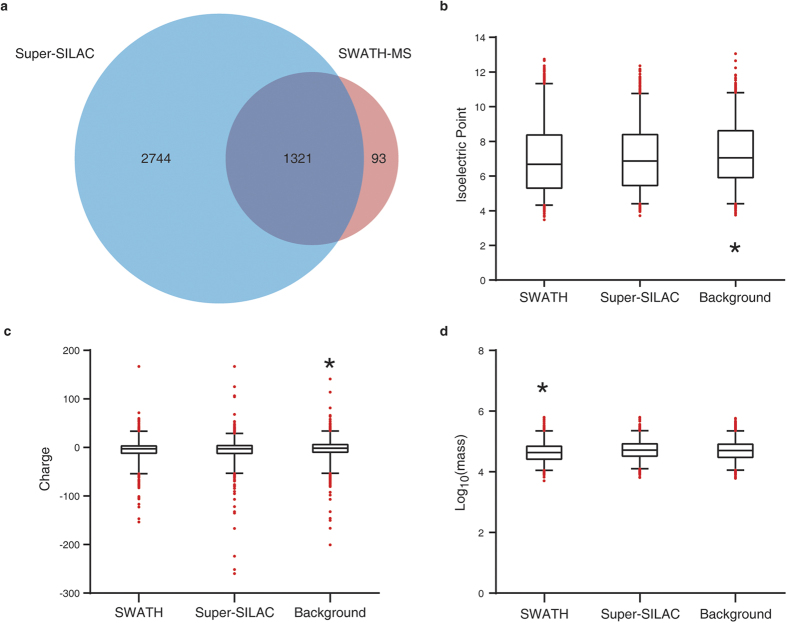
SWATH and super-SILAC-based MS identifications. (**a**) Venn diagram comparison of identification numbers. (**b**–**d**) Protein physical-chemical feature distributions, regarding isoelectric points (**b**) and charges at physiological conditions (**c**) and molecular weights (**d**). Data are presented with box-and-whisker plot, and 5% outliers are shown with red dots. A total of 1414 proteins were randomly sampled from the super-SILAC identifications and background human proteome, respectively, for the comparison with the SWATH MS identifications. *P < 0.01, as compared with any of the other groups, two-tailed Kolmogorov–Smirnov test.

We identified and quantified 1414 proteins across all 11 samples in the SWATH-MS analysis (Supplementary Table S3). While in the super-SILAC MS analysis on the pooled samples, 4065 proteins were quantified from both the normal and the NPC groups with protein FDR < 1% (Supplementary Table S4). Comparing the two methods, 1321 proteins were quantified in overlap (Fig. 1a). All MS raw data are available in iProX (accession number: IPX00080100).

We next focused on the physical-chemical characteristics of the SWATH-MS identified 1414 proteins, in comparison of the same amount (1414) of proteins randomly selected from super-SILAC identifications and the neXtProt PE1 proteins. Both MS methods preferentially detected proteins with lower isoelectric points (pI) (Fig. 1b) and higher charges (Fig. 1c) than the human proteome background. It is known that MS is keen on identifying more acidic proteins. In addition, more charges will lead to easier ionization of peptides, which is favorable for MS analyses. Hence, the above two features can be expected. Nonetheless, we found that SWATH-MS tended to identify smaller proteins with the median molecular weight (MW) of 43.7 kDa, significantly less than the super-SILAC MS identifications (median MW = 52.5 kDa) and the background PE1 proteins (median MW = 51.3 kDa) (Fig. 1d).

### Differentially expressed proteins in SWATH-MS

**Figure 2:**
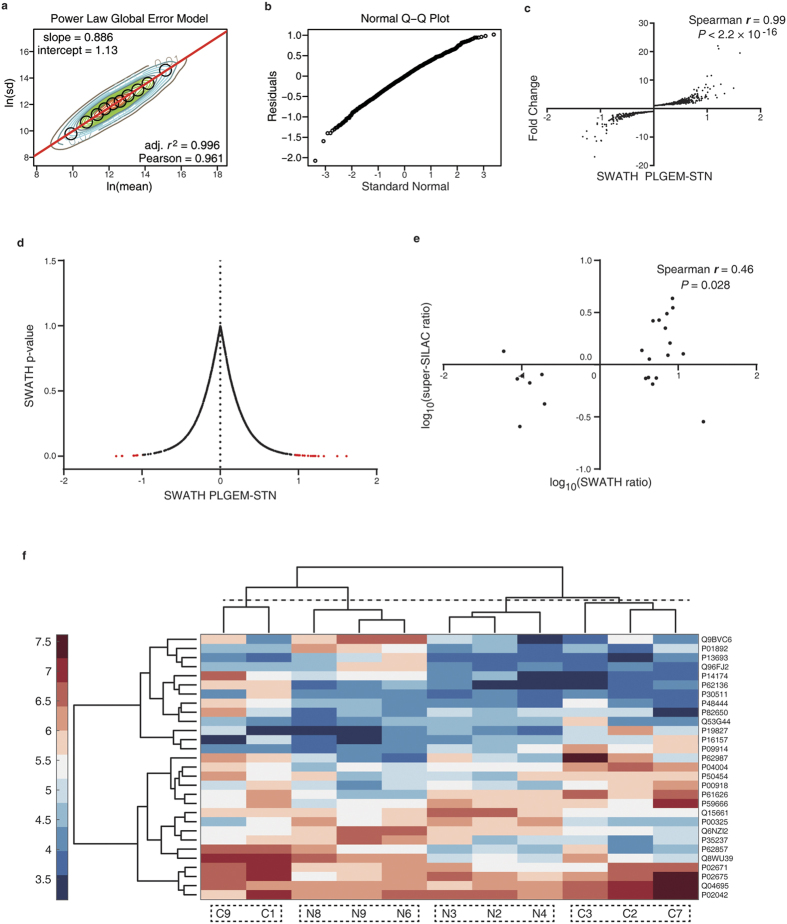
Determination of differentially expressed proteins in SWATH-MS. (**a**) Contour plots of ln (rowCV) versus ln (rowMean) scatter plots and the regression fitting analysis with PLGEM model. Black circles indicate the modeling points used to fit a PLGEM model. (**b**) Quantile-quantile (Q-Q) plot. (**c**) Correlation of signal-to-noise ratios (STN) and protein abundance fold changes. (**d**) A volcano plot showing the relationship between the PLGEM-STN and the p-value calculated by PLGEM. The DEPs are shown in red (*P* < 0.01). (**e**) DEP fold change correlation of SWATH-MS and super-SILAC MS analyses. (**f**) Cluster analysis using DEPs. C represents subjects with NPC, and N stands for normal subjects.

We next used the power law global error model (PLGEM) algorithm^26^ to determine differentially expressed proteins (DEPs). We and others have found that this method is of advantages to consider the global error while fitting the proteome abundance data for statistical tests^27^,^28^. We found that our SWATH-MS data could be well fitted by PLGEM, with the slope of 0.886 and adjusted *r*^2^ of 0.996 (Pearson *r* = 0.961) (Fig. 2a). The residuals between the modeled and the measured standard deviation (SD) generally followed normal distribution (Fig. 2b). These results suggested that PLGEM worked properly in dealing with the SWATH-MS data.

It has been shown that PLGEM provides a more powerful signal-to-noise (STN) ratio, especially in low-abundance protein evaluations, by incorporating the PLGEM-derived SD^26^,^27^. In this study, we observed that the PLGEM-STN was significantly correlated to the relative protein fold changes, with a Spearman *r* of 0.99 (Fig. 2c). Based on PLGEM-STN, we computed out 29 proteins as DEPs (*P* < 0.01), which took over ~2% of total proteins (Fig. 2d, Supplementary Table S2). These DEPs have certain reproducibility as quantified by super-SILAC and SWATH-MS (Fig. 2e). Such 29 DEPs could not completely separate the NPC tissues from the normal tissues per cluster analyses, potentially due to the small sample size (Fig. 2f). Despite this, we noted that 4 subgroups could be differentiated, and no NPC and normal tissues were clustered together (Fig. 2f).

### Common upstream regulator detected for SWATH and super-SILAC DEPs

**Figure 3:**
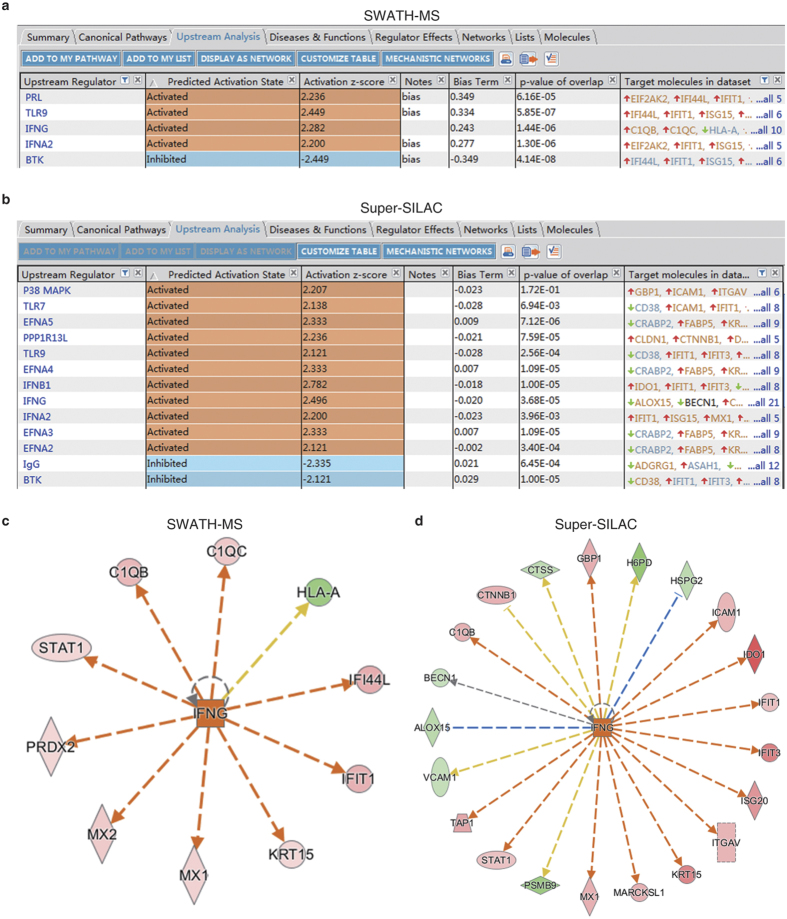
Upstream regulator analysis with IPA. (**a**,**b**) Upstream analysis of DEPs from SWATH-MS (**a**) and super-SILAC MS (**b**). Activated (z-score ≥ 2) and inhibited (z-score ≤ −2) upstream regulators are highlighted in orange and blue, respectively. (**c**,**d**) IFNG regulated proteins in SWATH-MS (**c**) and super-SILAC MS (**d**) analyses. Up-regulated and down-regulated proteins are highlighted in red and green, respectively, and the color depth is correlated to the fold change. Orange and blue dashed lines with arrows indicate indirect activation and inhibition, respectively. Yellow and gray dashed lines with arrows depict inconsistent effects and no prediction, respectively.

The upstream analysis module of Ingenuity Pathway Analysis (IPA) suggested that interferon gamma (IFNG) was the significantly unbiased upstream activator of SWATH-MS detected 10 DEPs (z-score = 2.282, *P* = 1.44 × 10^−6^; Fig. 3a). In the super-SILAC detected DEPs, consistent prediction of IFNG was computed out (Fig. 3b); in addition, other known viral infection relevant pathways, such as IFNA2, IFNB1 and TLR, were also deemed up-stream activators by IPA (Fig. 3b). Specific to the mechanistic network of IFNG, 5 common DEPs, such as STAT1 and IFIT1, were shared by both SWATH-MS (Fig. 3c) and super-SILAC MS (Fig. 3d). While other proteins were unique to methods; for example, PRDX2 was only detected in SWATH-MS (Fig. 3c). These results favored the complementary feature of different MS methods. In addition, such a similar upstream regulator prediction suggested that even we used different acquisition mode-based MS approaches (DDA or DIA), the bioinformatics implicated reproducibility, which favorably argued that our general research strategy was valid.

### SWATH spectra of CA2

**Figure 4:**
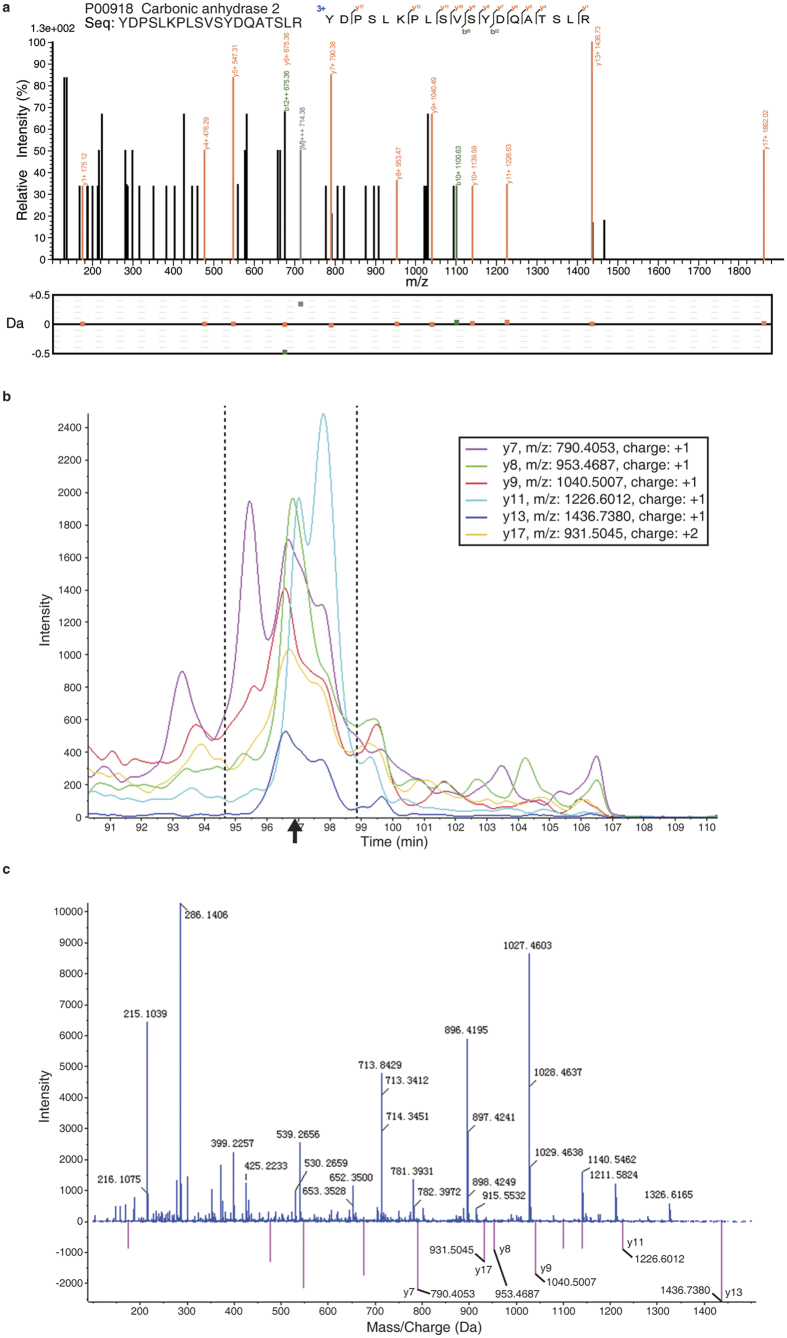
SWATH-MS spectra of CA2. (**a**) A shotgun spectrum of a CA2 unique peptide. The mass tolerance is shown for each detected fragment ion. (**b**) Retention time for the SWATH detected fragment ions. The black arrow indicates the retention time referenced by the parent ion detected in the shotgun mode. (**c**) The *m*/*z* distribution of SWATH detected fragment ions. The theoretical peaks of the CA2 ions were indicated by the vertical red lines, while the experimentally detected CA2 y-ions were specified.

Among the 29 DEPs detected by SWATH-MS, thirteen of them were also detected to be up-regulated in the super-SILAC MS analysis (Table 1). When applying the 1.5-fold threshold as referenced by the super-SILAC quantitation, seven proteins were left for further evaluation. We prioritized CA2 for further evaluation based on the following reasons: 1) CA2 had 4 unique peptides identified in the shotgun mode (Fig. 4a, and Supplementary Fig. S2), and the SWATH results could pass the manual spectra inspection; 2) Wu *et al*. previously reported that CA2 was up-regulated in the serum of NPC xenograft mice^29^; and 3) the CA2’s diagnostic power had not been reported in the NPC field.

As illustrated in Fig. 4, we showed an exclusively unique peptide spectrum of CA2 identified in the shotgun mode; it had 8 consecutive y-ions labeled with mass error less than 0.5 Da (Fig. 4a). When analyzed in the SWATH mode, 6 y-ions were found to have the retention time at ~97 min (black arrow, Fig. 4b), which was consistent with their parent-ion retention time as recorded in the shotgun mode. These 6 y-ions could be found in the MS2 spectrum of the SWATH analysis (Fig. 4c). These results proved that the SWATH identification of CA2 was of high confidence.

### CA2 has diagnostic value in NPC

**Figure 5:**
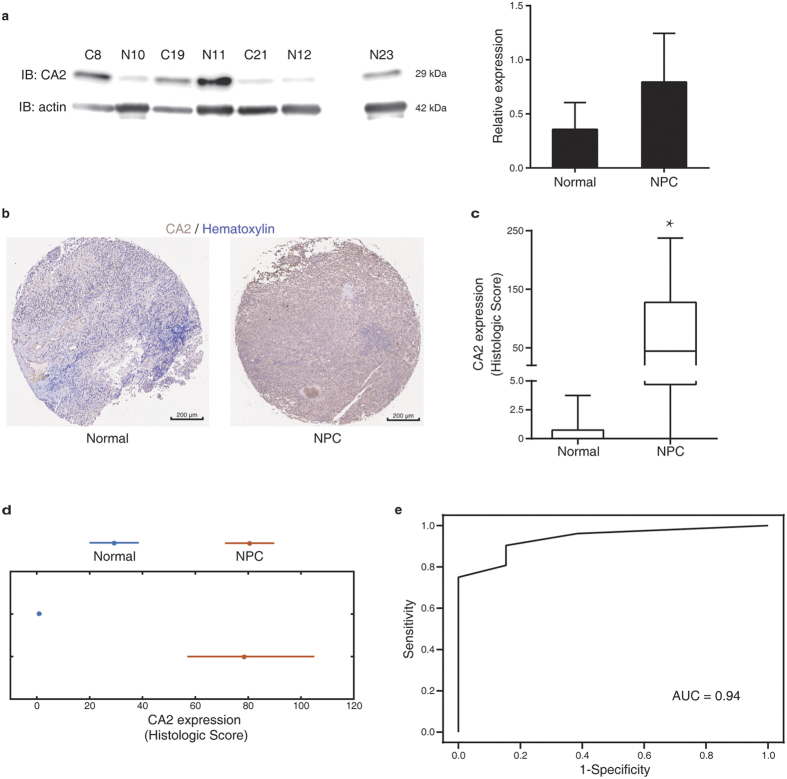
Diagnostic power verification of CA2. (**a**) Immunoblotting analysis on the CA2 expression in NPC and normal tissues. Data are shown as mean ± s.e.m. (**b**) Representative immunohistochemical images of CA2 staining in a tissue array of nasopharyngeal tissue from normal and NPC donors. (**c**) Statistical analysis of CA2 expressions. The Histologic Scores of each group are shown in a box-and-whisker plot. *P = 5.28 × 10^−6^, donor of normal n = 13, donor of NPC n = 52, two-tailed Kolmogorov–Smirnov test. (**d**) Bootstrap comparison of the CA2 expression. The star indicates the mean Histologic Score of each group, while the horizontal line depicts 95% confidence interval of the mean value acquired from 10000 resampled Histologic Scores. (**e**) The ROC curve generated from the Histologic Scores of all donors.

We next used more clinical samples to perform immunoblotting (IB) verification of CA2 changes. We found that although the NPC group tended to have more CA2 expression than the normal group, no significant difference was observed (Fig. 5a). The raw images of IB could be found in Supplementary Fig. S3. We reasoned that the sample size was not sufficient for the statistics of IB results. Thus, we employed a commercialized tissue microarray (NPC donor n = 52 and normal donor n = 13) to evaluate the diagnostic power of CA2. We could observe remarkably more CA2 expression in some NPC tissues, such as the example shown in Fig. 5b. But, we could still visually distinguish the within group variations; the immunohistochemistry (IHC) results of all tissue points could be found in Supplementary Fig. S1 and Table S5. Despite the variation, the KS-test results showed that the CA2 Histologic Scores of the NPC tissues were significantly higher than the normal tissues (Fig. 5c). We noted that the sample size was considerably different between the two groups. As such, we used bootstrap analysis to further verify the conclusion. We found that post 10000 times bootstrap resampling from each group, the 95% confidence intervals of the two groups were not overlapped, suggesting that the mean values of the two groups were statistically different (Fig. 5d). With receiver operating characteristic (ROC) analysis on the Histologic Score, we found that CA2 showed high diagnostic power with the Area Under Curve (AUC) of 0.94 (Fig. 5e).

## Discussion

In this study, we demonstrated that SWATH-MS in the DIA mode worked properly in the NPC biomarker discovery with a unique feature to preferentially detect proteins with low MWs. With such an aide, we have identified and justified that CA2 is a potentially new diagnostic biomarker that has high statistical power to differentiate NPC from normal tissues.

In our past work, we have found that human cells are preferentially translating shorter mRNAs, and when considering the mRNA length, translating mRNAs and proteins are highly correlated in their abundances^30^. We have further proposed a computational model to justify that such length-dependence translation preference is a survival strategy of human cells to maintain a functional proteome by avoiding erroneous protein products^31^. These findings suggest that the stoichiometry of translating mRNA into protein is correlated to the mRNA length^30^,^31^. A recent study from Huang *et al*. have evidenced that SWATH-MS signal intensities have precisely linear correlation to the sample loading abundances in a label-free analysis^32^. It is known that SWATH scanning is generally referenced by the shotgun library, which is usually based on single-injection MS analysis. In such a scenario, proteins with higher molar concentrations will have a higher probability to be identified by the shotgun MS. In sum, SWATH-MS tends to focus on the fragment ions derived from protein products with less amino acid length, while it has outstanding quantitation precision. Such a unique feature of SWATH-MS should partially explain its complementary feature to other MS approaches, such as super-SILAC results demonstrated in this study. It should not be a surprise to see its capacity in identifying new biomarkers.

In human, the carbonic anhydrase family has 16 enzymes that catalyze the reversible reaction from carbon dioxide and water to bicarbonate and protons. They have diversified associations with cancer, autoimmune disease and viral infection^33^,^34^. Viikila *et al*. have found that CA2 and CA12 have prognostic power in colorectal carcinomas^35^; and Kurono *et al*. have reported that CA2 expression in breast cancer is significantly higher than normal tissues^36^. It is known that solid tumors are featured by the tumor microenvironment, with aberrant activation of numerous immune cells^28^. Interestingly, CA2 and other carbonic anhydrases can cause autoimmune reaction via activating mast cells^37^ and plasma cells^38^,^39^. Thus, the diagnostic power of CA2 on NPC has comparable evidence from other solid tumor types, and it potentially reflects the inflaming tumor microenvironment.

Furthermore, we demonstrated that the DEPs detected by SWATH-MS had significantly common regulator of IFNG, which implicated the anti-viral response in NPC subjects. IFNG is an essential cytokine produced by both innate and adaptive immune cells, which is highly active to fight against viral, certain bacteria and protozoal infections. The IFNG level is to rise post primary EBV infection in humans^40^; while along with the increased EBV viral load, the serum IFNG level increases in NPC patients^41^. Such rationales help to argue the biological relevance of the SWATH-MS DEPs to NPC or EBV infection. Favorably, among these DEPs, other groups have proven VTN^42^,^43^ and MIF^44^,^45^ as potential NPC biomarkers. Therefore, as the viral response contributes to the NPC-associated inflammation, these findings suggest that proteins participated such a bioprocess constitute a resource of potential NPC diagnosis biomarkers.

## Methods

### Human nasopharyngeal tissue samples

The nasopharyngeal tissue samples were acquired from The First Affiliated Hospital of Jinan University via biopsy. The scientific and ethics review committees of Jinan University approved this study, and written informed consents were obtained from all of the study participants. All methods were performed in accordance with the relevant guidelines and regulations.

### Cell culture

Human cell line CNE-1 and CNE-2 were acquired from American Type Culture Collections (ATCC, Rockville, MD). All cells were maintained in the complete Dulbecco’s modified Eagle’s medium (DMEM), supplemented with 10% fetal bovine serum (FBS), 1% penicillin/streptomycin, 10 μg/mL ciprofloxacin.

### SILAC labeling

CNE-1 and CNE-2 were subjected to SILAC labeling as we previously described^28^,^46^. In brief, cells were cultured in the heavy SILAC medium, DMEM containing 73 mg/L ^13^C_6_^15^N_2_-L-lysine (Lys8) and 42 mg/L^13^C_6_^15^N_4_-L-arginine (Arg10) (Cambridge Isotope Laboratories, Andover, MA, USA), supplemented with 10% dialytic FBS (Life Technologies), 1% pen/strep and various forms of essential amino acids (Cambridge). After at least 8 passages, cells were lysed, and a pooled cell lysate was used for the spike-in standard for the subsequent super-SILAC-based shotgun MS analysis as developed by Mann’s group^47^.

### Protein extraction and digestion

Post-biopsy, tissue samples were immediately treated with 1% SDS lysis buffer (Beyotime, Nanjing, China), supplemented with 1 mM phenylmethanesulfonyl fluoride (PMSF), and 2% (v:v) protease inhibitor (Roche, Shanghai, China), followed by grounding extraction in liquid nitrogen. The tissue lysate was sonicated and centrifuged at 17,000 × g for 30 min. Supernatants were collected, and the protein concentration was determined by a BCA kit (ThermoFisher Scientific, Shanghai, China). Regarding cell sample lysis, we followed our reported procedure^48^.

We employed in-solution protein digestion with a filter-aided sample preparation (FASP) method^49^, as we described previously^48^. Briefly, samples were subjected to reduction (8 M urea and 50 mM DTT at 37 °C, 1 h) and alkylation (100 mM IAA, at room temperature, 30 min) in the 30 kDa ultracentrifugal filters (Sartorius Stedim Biotech, Shanghai, China). After 15 min centrifugation (12,000 × *g*, 4 °C), two sequential buffer changes were performed using 8 M urea and 50 mM NH_4_HCO_3_, respectively. Trypsin was then added into the filter at a mass ratio of 1:30 for 8 h, at 37 °C. Peptides were collected by centrifugation at 12,000 × g, 4 °C, 15 min.

### Super-SILAC-based shotgun mass spectrometry

Tissue protein extracts were mixed with the SILAC labeled spike-in standard at a 1:1 mass ratio prior to the in-solution digestion. Peptides were fractionated with an approach using SAX StageTip and C18 StageTips, and 6 peptide fractions were collected by using serial elution buffers with pH values of 11, 8, 6, 5, 4 and 3^46^,^47^. Peptides were then analyzed with a TripleTOF^®^ 5600 MS (5600 MS; AB SCIEX, Framingham, CA, USA) as we previously described^28^,^46^. MS parameters: spray voltage, 2.3 kV; interface heater temperature, 120 °C; scan range, 350–1500 m/z; mass tolerance, 50 mDa; resolution, >30 k fwhm; information-dependent acquisition (IDA) MS/MS scans, applied; maximum number of candidate ions per cycle, 40; charge state, 2–4 and >200 cps; dynamic exclusion, applied; cooccurrence, 1; and duration, 20 s.

The MS raw data were searched with ProteinPilot^®^ v4.5 (Revision: 1656; Paragon Algorithm: 4.5.0.0, 1654) against the Human Swiss-Prot database (Feb, 2015). Search parameters included, Sample Type: SILAC (Lys + 8, Arg + 10); Cys Alkylation: Iodoacetamide; Digestion: Trypsin; Instrument: TripleTOF 5600; Species: Homo sapiens; ID Focus: Biological modifications; Search Effort: Thorough ID; Detected Protein Threshold: >10% Conf.; Special Factors: none. The global FDR from Fit at protein level was set to 1%.

### SWATH mass spectrometry

To generate the ion library for the SWATH-MS analysis, peptides from different nasopharyngeal tissue samples were pooled, and analyzed in a shotgun model with a TripleTOF^®^ 5600 MS (AB). In detail, peptides were analyzed in the high sensitivity IDA mode; precursor ion selection range was 350~1500 *m*/*z*, using 0.25 s accumulation. For each precursor ion with 50 ms minimum accumulation time in the range of 100–1500 *m*/*z*, the maximum precursor number per cycle was set to 40. Dynamic exclusion was applied.

Next, peptides of each tissue samples were individually analyzed in the SWATH mode^18^. Specifically, the 14* m*/*z* precursor isolation window was used for the consecutive data-independent acquisition across the range of 400~1150 *m*/*z*. The accumulation time was set to 50 ms, and the total cycle time was ~3.2 s.

The SWATH-MS raw data were searched by ProteinPilot with the identical parameters to those used for the SILAC searches, except that the Sample Type was set as Identification. The resulting group file was loaded into PeakView^®^ v1.2.0.3 (SWATH^TM^ Acquisition MicroApp: v1.0.0.653). The Maximum Number of Protein to Import was set as the number of proteins identified at 1% global FDR from Fit. Other parameters included: The Ion Library Mass Tolerance, 75 ppm; XIC Extraction Window, 20 min; and XIC width, 75 ppm. The ion library was filtered by 6 peptides per protein and 6 fragment ions per peptide with a Peptide Confidence of 85 per protein. Variably modified or shared peptides were excluded.

### Physical and Chemical Features of Proteins

The amino acid sequence and mass were obtained from Swiss-Prot human protein database. The isoelectric point (pI) and the charge at physiological conditions (pH = 7.4) of each protein were calculated by MATLAB bioinformatics toolbox (MathWorks, Natick, MA, USA).

### Ingenuity Pathway Analysis

We performed core analysis of IPA for fold changes of DEPs in each MS method as we describe previously^28^,^50^. The Upstream Analysis was used to find out upstream regulators of DEPs with activation/inhibition status predictions^51^.

### Immunoblotting analysis

IB was performed as we previously described^46^,^48^,^51^. Antibodies used were rabbit anti-CA2 pAb (1:2000, PROTEINTECH GROUP, INC., China) and HRP-conjugated goat anti-rabbit mAb (1:2000, Bioworld).

### Immunohistochemistry

A commercial tissue array (array number: NPC1503, Super Biotek, Shanghai, China) was used to analyze the diagnostic power of CA2. IHC was performed by using rabbit anti-CA2 pAb (1:100, Sino Biological Inc., Beijing, China) and the SuperPicture™ 3rd Gen IHC Detection Kit (Thermo). The tissue array was incubated with the primary antibody, followed by the visualization with DAB staining and hematoxylin counter-staining. The staining intensity was scored as 0 (negative), 1 (weak), 2 (moderate) and 3 (strong), independently evaluated by at least two professional pathologists. A Histologic Score (intensity score × percentage of stained cell) was used to evaluate the CA2 expression.

### Statistics

DEPs were determined by using the *R* package PLGEM^27^,^28^, and the statistical significance was accepted when *P* < 0.01. All of the correlation analyses were shown with Spearman *r*. Histologic Score of IHC results were examined by the unpaired Kolmogorov–Smirnov test (KS-test), using GraphPad Prism version 6.02 (GraphPad Software, Inc., San Diego, CA, USA), which *P* < 0.05 was deemed significantly different. In addition, we employed the bootstrap analysis to compensate the variation from the different sample sizes provided by the tissue microarray. The 10000 bootstrap resampling was performed for each group to statistically compare the Histologic Scores of the NPC and the normal groups^46^,^48^. Both bootstrap and cluster analyses were generated by MATLAB software version R2016a. The ROC curve and the AUC were generated and computed by GraphPad Prism.

## Additional Information

**How to cite this article**: Luo, Y. *et al*. SWATH-based proteomics identified carbonic anhydrase 2 as a potential diagnosis biomarker for nasopharyngeal carcinoma. *Sci. Rep.* **7**, 41191; doi: 10.1038/srep41191 (2017).

**Publisher's note:** Springer Nature remains neutral with regard to jurisdictional claims in published maps and institutional affiliations.

## Supplementary Material

Supplementary Materials

Supplementary Table S3

Supplementary Table S4

Supplementary Table S5

## Figures and Tables

**Table 1:** Differentially expressed proteins of SWATH-MS referenced by super-SILAC MS.

Swiss-Prot ID	Protein name	NPC/normal ratio in super-SILAC	NPC/normal average ratio in SWATH	SWATH *P*-value	HGNC Gene name
P16157	Ankyrin-1	4.32	8.48	8.64 × 10^−3^	*ANK1*
P04004	Vitronectin	3.51	8.56	9.53 × 10^−4^	*VTN*
Q04695	Keratin, type I cytoskeletal 17	3.09	7.21	1.10 × 10^−4^	*KRT17*
P00918	Carbonic anhydrase 2	2.67	5.71	7.05 × 10^−3^	*CA2*
P50454	Serpin H1	2.64	4.77	8.84 × 10^−3^	*SERPINH1*
P09914	Interferon-induced protein with tetratricopeptide repeats 1	2.24	6.88	5.39 × 10^−3^	*IFIT1*
P14174	Macrophage migration inhibitory factor	1.61	7.88	3.24 × 10^−3^	*MIF*
Q8WU39	Marginal zone B- and B1-cell-specific protein	1.37	3.44	6.74 × 10^−3^	*MZB1*
P30511	HLA class I histocompatibility antigen, alpha chain F	1.3	11.4	7.40 × 10^−3^	*HLA-F*
P48444	Coatomer subunit delta	1.27	11.61	4.53 × 10^−3^	*ARCN1*
P82650	28 S ribosomal protein S22, mitochondrial	1.23	7.4	6.93 × 10^−3^	*MRPS22*
P62136	Serine/threonine-protein phosphatase PP1-alpha catalytic subunit	1.19	22.04	1.61 × 10^−3^	*PPP1CA*
P62857	40 S ribosomal protein S28	1.13	4.29	9.40 × 10^−3^	*RPS28*
