# Supplementary material for: SWATH-based proteomics identified carbonic anhydrase 2 as a potential diagnosis biomarker for nasopharyngeal carcinoma
Source: Sci Rep. 2017 Jan 24;7:41191. doi: 10.1038/srep41191 (PMC5259699; doi:10.1038/srep41191)
Supplement: Supplementary Materials [file srep41191-s1.pdf]

# SWATH-based proteomics identified carbonic anhydrase 2 as a potential diagnosis biomarker for nasopharyngeal carcinoma

Yanzhang Luo, Tin-Seak Mok, Xiuxian Lin, Wanling Zhang, Yizhi Cui, Jiahui Guo, Xing Chen,

Tao Zhang*, Tong Wang*

# Supplementary Information

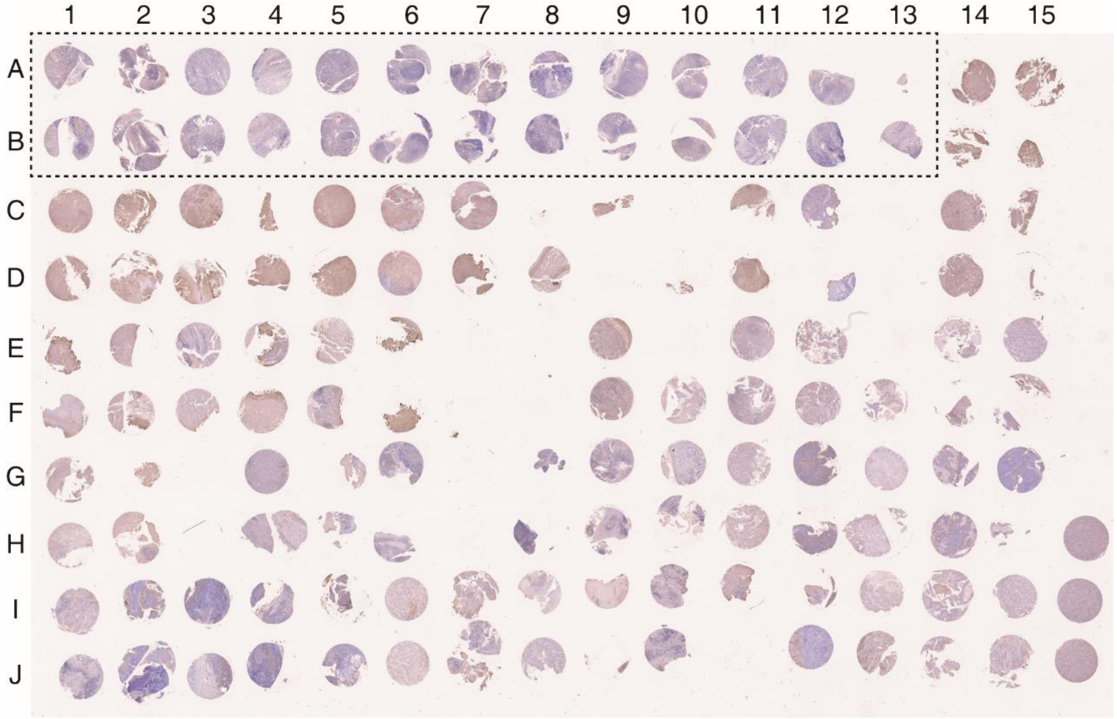


**Supplementary Figure S1. Raw image of tissue microarray.** Normal samples are shown in the dashed square box. Each sample has a replicate at the next vertical row. For example, samples in line B are replicates of line A at each donor tissue point. (NPC donor n = 52 and normal donor n = 13).

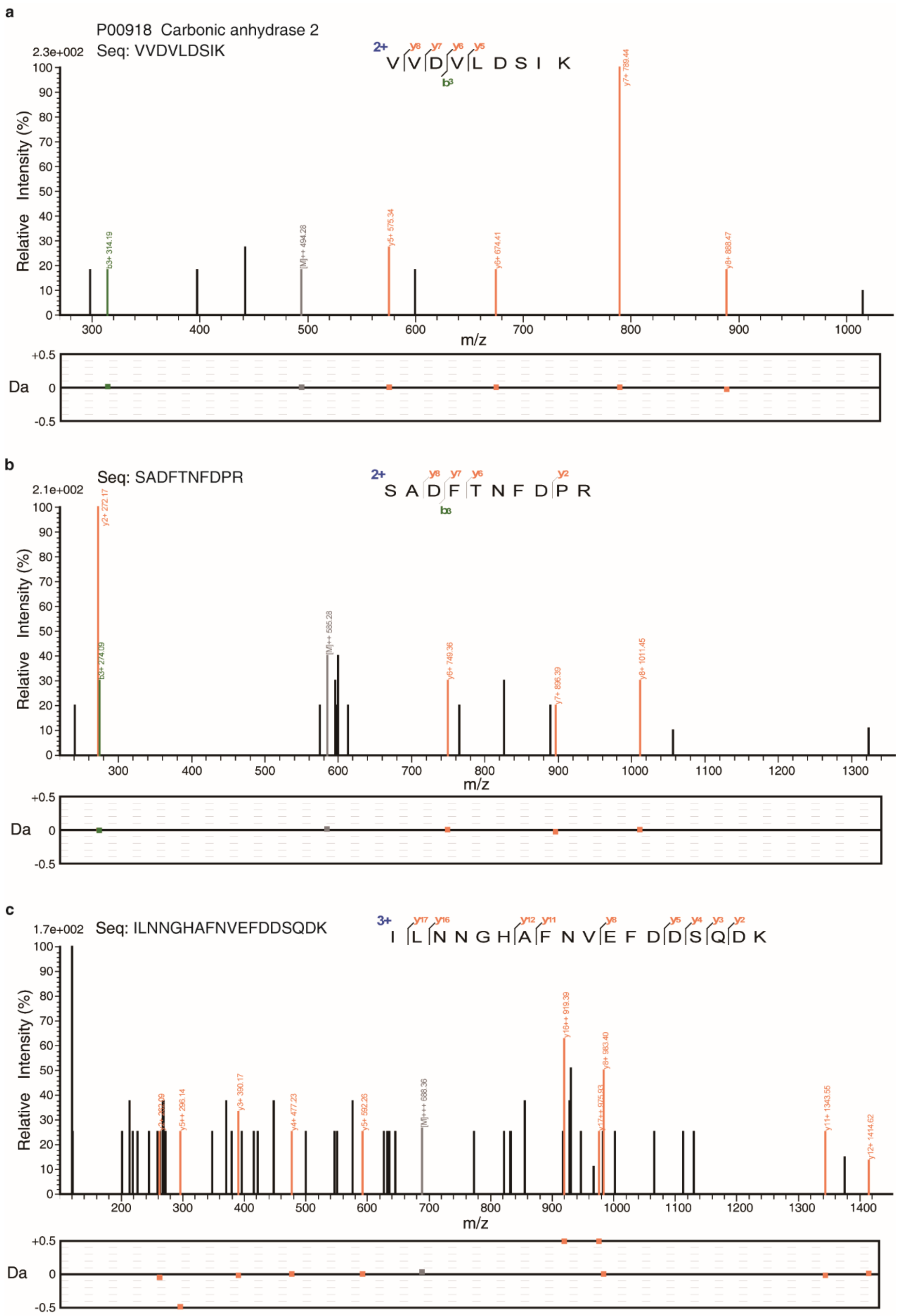


**Supplementary Figure S2. MS spectra of CA2 peptides detected in the shotgun library construction identification.**

a

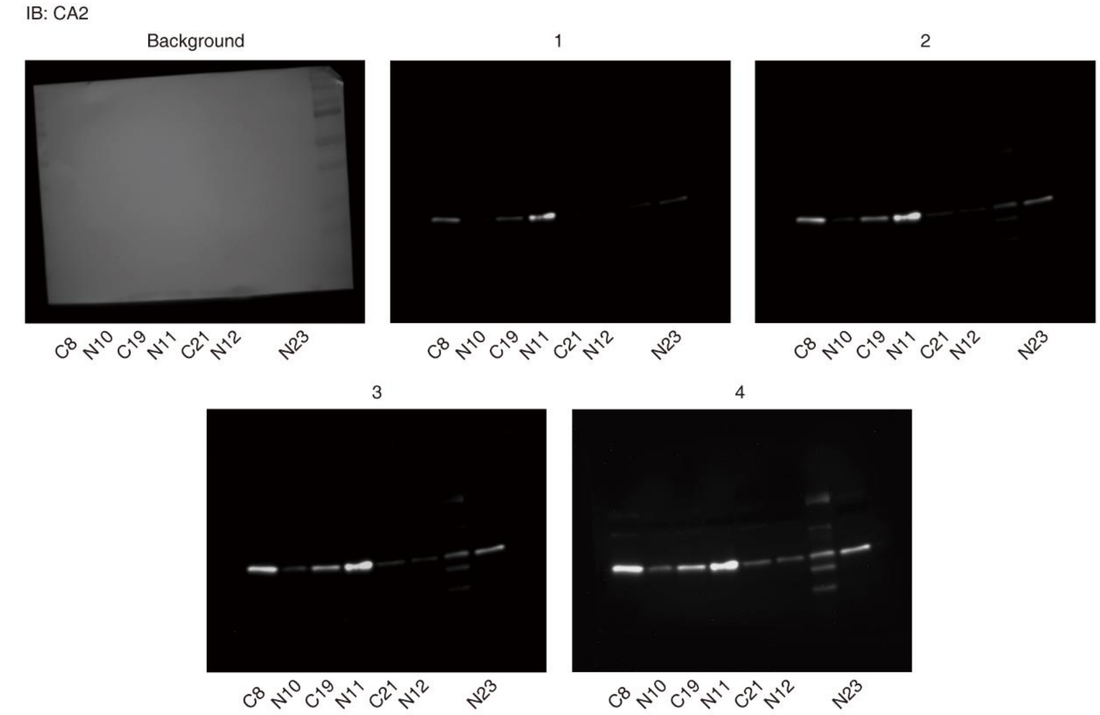


b

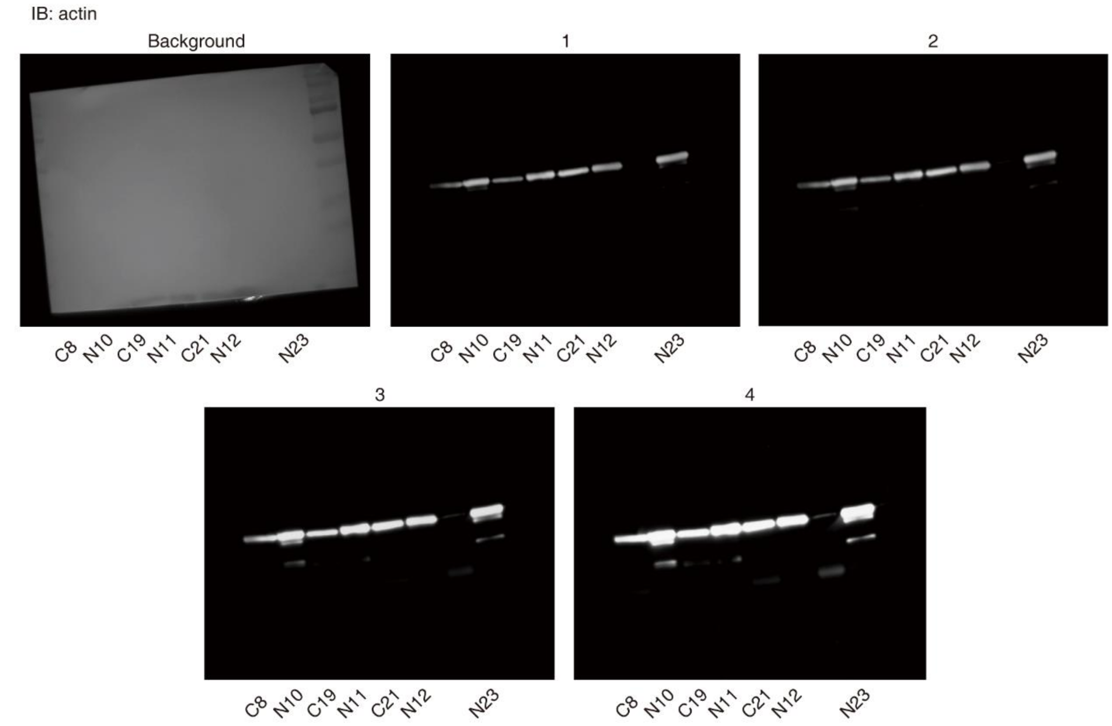


**Supplementary Figure S3. Raw image of the immunoblotting analysis on CA2 (a) and actin (b) with multiple exposures.**

**Supplementary Table S1.** Donor feature summary.

| | Total | Gender | | Age | |
|---|---|---|---|---|---|
| | | **Male** | **Female** | **< 50** | **≥50** |
| **NPC** | 11 | 8 | 3 | 6 | 5 |
| **Normal** | 13 | 9 | 4 | 11 | 2 |
| ***P*** | | 1.0000 | | 0.1819 | |

**Supplementary Table S2.** Differentially expressed proteins of SWATH-MS determined by PLGEM analysis.

| No. | Swiss-Prot ID | Protein name | NPC/normal abundance ratio | PLGEM STN | PLGEM P-value | HGNC gene name |
|---|---|---|---|---|---|---|
| 1 | P62136 | Serine/threonine-protein phosphatase PP1-alpha catalytic subunit | 22.04 | 1.2 | $1.61 \times 10^{3}$ | *PPP1CA* |
| 2 | P19827 | Inter-alpha-trypsin inhibitor heavy chain H1 | 21.02 | 1.22 | $1.43 \times 10^{3}$ | *ITIH1* |
| 3 | P62987 | Ubiquitin-60S ribosomal protein L40 | 19.52 | 1.61 | $3.96 \times 10^{5}$ | *UBA52* |
| 4 | P48444 | Coatomer subunit delta | 11.61 | 1.08 | $4.53 \times 10^{3}$ | *ARCN1* |
| 5 | P30511 | HLA class I histocompatibility antigen, alpha chain F | 11.4 | 1 | $7.40 \times 10^{3}$ | *HLA-F* |
| 6 | P04004 | Vitronectin | 8.56 | 1.26 | $9.53 \times 10^{4}$ | *VTN* |
| 7 | P16157 | Ankyrin-1 | 8.48 | 0.98 | $8.64 \times 10^{3}$ | *ANK1* |
| 8 | P14174 | Macrophage migration inhibitory factor | 7.88 | 1.12 | $3.24 \times 10^{3}$ | *MIF* |
| 9 | P82650 | 28S ribosomal protein S22, mitochondrial | 7.4 | 1.01 | $6.93 \times 10^{3}$ | *MRPS22* |
| 10 | Q53G44 | Interferon-induced protein 44-like | 7.36 | 0.96 | $9.87 \times 10^{3}$ | *IFI44L* |
| 11 | Q04695 | Keratin, type I cytoskeletal 17 | 7.21 | 1.5 | $1.10 \times 10^{4}$ | *KRT17* |
| 12 | P09914 | Interferon-induced protein with tetratricopeptide repeats 1 | 6.88 | 1.05 | $5.39 \times 10^{3}$ | *IFIT1* |
| 13 | P00918 | Carbonic anhydrase 2 | 5.71 | 1.01 | $7.05 \times 10^{3}$ | *CA2* |
| 14 | P02675 | Fibrinogen beta chain | 5.27 | 1.33 | $5.17 \times 10^{4}$ | *FGB* |
| 15 | P50454 | Serpin H1 | 4.77 | 0.97 | $8.84 \times 10^{3}$ | *SERPINH1* |
| 16 | P02671 | Fibrinogen alpha chain | 4.73 | 1.16 | $2.36 \times 10^{3}$ | *FGA* |
| 17 | P62857 | 40S ribosomal protein | 4.29 | 0.96 | $9.40 \times 10^{3}$ | *RPS28* |

| | | | | | | |
|---|---|---|---|---|---|---|
| | | S28 | | | | |
| 18 | P61626 | Lysozyme C | 4.17 | 0.99 | $8.14 \times 10^3$ | *LYZ* |
| 19 | P02042 | Hemoglobin subunit delta | 3.85 | 1.18 | $1.89 \times 10^3$ | *HBD* |
| 20 | P59666 | Neutrophil defensin 3 | 3.83 | 0.96 | $9.58 \times 10^3$ | *DEFA3* |
| 21 | Q8WU39 | Marginal zone B- and B1-cell-specific protein | 3.44 | 1.02 | $6.74 \times 10^3$ | *MZB1* |
| 22 | Q15661 | Tryptase alpha/beta-1 | 0.2 | -1.06 | $6.69 \times 10^3$ | *TPSAB1* |
| 23 | P35237 | Serpin B6 | 0.18 | -1.06 | $6.71 \times 10^3$ | *SERPINB6* |
| 24 | Q6NZI2 | Polymerase I and transcript release factor | 0.13 | -1.26 | $1.36 \times 10^3$ | *PTRF* |
| 25 | P01892 | HLA class I histocompatibility antigen, A-2 alpha chain | 0.1 | -1.1 | $4.86 \times 10^3$ | *HLA-A* |
| 26 | Q9BVC6 | Transmembrane protein 109 | 0.1 | -1.33 | $7.29 \times 10^4$ | *TMEM109* |
| 27 | Q96FJ2 | Dynein light chain 2, cytoplasmic | 0.09 | -1.07 | $6.03 \times 10^3$ | *DYNLL2* |
| 28 | P00325 | Alcohol dehydrogenase 1B | 0.09 | -1.25 | $1.43 \times 10^3$ | *ADH1B* |
| 29 | P13693 | Translationally-controlled tumor protein | 0.06 | -1.11 | $4.61 \times 10^3$ | *TPT1* |

**Supplementary Table S3.** Identification and quantitation results of SWATH MS.
(Separate excel file)

**Supplementary Table S4.** Identification and quantitation results of super-SILAC MS.
(Separate excel file)

**Supplementary Table S5.** Clinicopathological information and CA2 staining results of the tissue microarray analysis.
(Separate excel file)
